# BioVars - A bioclimatic dataset for Europe based on a large regional climate ensemble for periods in 1971–2098

**DOI:** 10.1038/s41597-025-04507-w

**Published:** 2025-02-05

**Authors:** Anne Reichmuth, Oldrich Rakovec, Friedrich Boeing, Sebastian Müller, Luis Samaniego, Andreas Marx, Hanna Komischke, Andreas Schmidt, Daniel Doktor

**Affiliations:** 1 Helmholtz-Centre for Environmental Research - UFZ, Department Remote Sensing, Leipzig, 04318 Germany; 2 Remote Sensing Centre for Earth System Research, RSC4Earth, Leipzig University, Leipzig, Germany; 3 Helmholtz-Centre for Environmental Research - UFZ, Department Computational Hydrosystems, Leipzig, 04318 Germany; 4 Faculty of Environmental Sciences, Czech University of Life Sciences Prague, Praha, Suchdol 16500 Czech Republic; 5 University of Potsdam, Institute of Environmental Science and Geography, Potsdam, Germany; 6 Leipzig University, Institute for Earth System Science and Remote Sensing, Leipzig, 04103 Germany; 7 German Centre for Integrative Biodiversity Research (iDiv) Halle-Jena-Leipzig, Leipzig, 04103 Germany

**Keywords:** Projection and prediction, Ecological modelling

## Abstract

Ongoing ecological research is concerned with analysing climate-induced changes in species distribution. For this purpose, the projection must have high-quality bioclimatic variables from historical and future climatic periods for the projection. To date, there are many global bioclimatic variables on this topic. Nevertheless, a consistent dataset with identical model variables from historic and projected periods is rare. We present 26 bioclimatic variables that are calculated based on a large ensemble consisting of 70 bias-adjusted GCM-RCM simulations for 1971–2098. Both, the historic and the projection periods were calculated using the same models to ensure consistency between the periods. The variables are validated against E-OBS observations from which we calculated the same bioclimatic variables. For projection periods we chose 20 year ranges between 2021–2098. Here, we offer two versions of them: (1) variables separated into RCP 2.6, 4.5 and 8.5, including percentiles among the realisations and within the RCPs; and (2) variables per realisation separately. We then extracted the temporal 5th, 50th and 95th percentile per period as representing values.

## Background & Summary

Current ecological research deals with the subject of analysing climate-induced changes in species distribution. In recent years the research interest concerning various ecological topics greatly increased^1–3^. Common knowledge is that the distribution of vegetation highly depends on the climate conditions^4,5^. To quantify this climate induced forcing, direct gradients in the form of bioclimatic variables are widely used as predictors as they have physiological relevance to species^6^. A vast amount of global bioclimatic variables are already available with various spatial and temporal resolutions, based on different source data. Data from historic periods are often generated using direct observations such as station data in conjunction with indirect observations, for instance remote sensing data (*WorldClim*^7,8^) or based on remote sensing data alone (*MERRAclim*^9^). Others were derived from the before mentioned *WorldClim* in combination with CRU^10^ data (*CliMond*^11,12^) or are either based on ERA-Interim^13^ in combination with station data (*CHELSA*^14^) or ERA5 reanalysis data^15^ (*BIOCLIMATE*^16,17^). A very different approach is used to derive past (Holocene), present and future projections (*ecoClimate*^18^) through simulation data from CMIP5 (Coupled Modeling Intercomparison Projects)^19^ and PMIP3 (Paleoclimate Modeling Intercomparison Projects)^20^. Similarly, simulation data from GCMs (Global Circulation Models) are utilised to derive past conditions for the late Quaternary^21^ (last 120,000 years).

Nearly all data sources mentioned provide historic data but also future projections (*WorldClim*, *CliMond*, *BIOCLIMATE*, *ecoClimate*). However, solely *ecoClimate* uses identical source data for all time periods. Especially *WoldClim* data^7,8^ can be considered as standard predictor variables for species distribution models. The data basis for *WoldClim*’s future and historic periods are different. For the historic period 1970 to 2000 it is gridded station data in conjunction with remote sensing products. For the future projections it is CMIP6^22^ GCMs (General Circulation Models or Global Circulation Models). This can introduce a climate model bias, which can be minimised through bias-adjustment in order to correct the statistics of the projections during a historic time period. Nevertheless, the provided data of the historic time periods differ in methodology from the projection data. This introduces additional bias as both datasets exhibit a different characteristic which will be passed on to following applications. The CMIP6 GCMs carry a spatial resolution of 20 km to 260 km^23^ depending on the model used. On the other hand, CMIP5 simulations with RCM for Europe in the framework of EURO-CORDEX^24–26^ offer a resolution of ~12.5 km and are to date regional climate projections with the highest resolution in large ensembles.

We present 26 bioclimatic variables with an European extent for the historic 1971 to 2000 and the projection period 2021 to 2098, both based on regionalised CMIP5 simulation data from EURO-CORDEX and ReKlies-DE^27^ from within the Helmholtz-Initiative Climate Adaptation and Mitigation (HI-CAM) initiative^28^. The input climate variables are bias-adjusted using E-OBS data (version v20.0e)^29^ and spatially disaggregated. The extracted historic bioclimatic data are validated against equivalent E-OBS based variables. Our variables allow training and projection on the same simulation data and thus same characteristics, which lessens the bias for further applications such as species distribution modelling.

## Methods

### Data source

Regional Climate simulations serve as input for the calculation of bioclimatic variables. These climate simulations were bias-corrected and disaggregated within the Helmholtz-Initiative (HI-CAM)^28^ and consist of regionalised CMIP5 simulations from EURO-CORDEX and ReKlies-DE^27^. They encompass data based on 11 Regional Climate Model (RCM) downscaling simulations as output from 10 different Global Climate Models (GCM). These simulations consist of 88 realisations that can be assigned to 3 different Representative Concentration Pathways (RCP) 2.6, 4.5 and 8.5 defined by Intergovernmental Panel on Climate Change (IPCC)^30^. Realisations with GCM *CNRM-CM5* and/or RCM *HIRHAM5* involved were omitted according to errata^31^ and (https://www.euro-cordex.net/078730/index.php.en, last access: 20th June 2024) at the time of simulation creation. This leads to omitting 18 realisations and therefore leading to 70 valid realisations, (final realisations listed under Table 1). Thereof 18 realisations are counted as RCP 2.6, 13 realisations as RCP 4.5 and 39 realisations as RCP 8.5.

**Table 1:** Overview of all considered realisations within HI-CAM^28^ EURO-CORDEX^24–26^ CMIP5^19^ project (Information and data can be retrieved from https://aims2.llnl.gov/search/cmip5/https://aims2.llnl.gov/search/cmip5/).

rcp	inst	gcm	inst.rcm	realisation	met_id
RCP 2.6	ICHEC	EC-EARTH	CLMcom-CCLM4–8–17	r12i1p1	met_11
			GERICS-REMO2015	r12i1p1	met_12
			KNMI-RACMO22E	r12i1p1	met_13
			SMHI-RCA4	r12i1p1	met_14
	IPSL	IPSL-CM5A-LR	GERICS-REMO2015	r1i1p1	met_32
	MIROC	MIROC5	CLMcom-CCLM4-8-17	r1i1p1	met_38
			GERICS-REMO2015	r1i1p1	met_39
	MOHC	HadGEM2-ES	GERICS-REMO2015	r1i1p1	met_42
			ICTP-RegCM4-6	r1i1p1	met_43
			KNMI-RACMO22E	r1i1p1	met_44
			SMHI-RCA4	r1i1p1	met_45
	MPI-M	MPI-ESM-LR	CLMcom-BTU-CCLM4-8-17	r1i1p1	met_59
			MPI-CSC-REMO2009	r1i1p1	met_60
				r2i1p1	met_62
			SMHI-RCA4	r1i1p1	met_61
	NCC	NorESM1-M	GERICS-REMO2015	r1i1p1	met_79
			SMHI-RCA4	r1i1p1	met_80
	NOAA-GFDL	GFDL-ESM2G	GERICS-REMO2015	r1i1p1	met_88
RCP 4.5	ICHEC	EC-EARTH	CLMcom-CCLM4-8-17	r12i1p1	met_16
			KNMI-RACMO22E	r12i1p1	met_17
				r1i1p1	met_19
			SMHI-RCA4	r12i1p1	met_18
	IPSL	IPSL-CM5A-MR	IPSL-WRF381P	r1i1p1	met_33
			SMHI-RCA4	r1i1p1	met_34
	MOHC	HadGEM2-ES	CLMcom-CCLM4-8-17	r1i1p1	met_46
			KNMI-RACMO22E	r1i1p1	met_48
			SMHI-RCA4	r1i1p1	met_49
	MPI-M	MPI-ESM-LR	CLMcom-CCLM4-8-17	r1i1p1	met_63
			MPI-CSC-REMO2009	r1i1p1	met_64
				r2i1p1	met_66
			SMHI-RCA4	r1i1p1	met_65
RCP 8.5	CCCma	CanESM2	CLMcom-CCLM4-8-17	r1i1p1	met_01
			GERICS-REMO2015	r1i1p1	met_02
	ICHEC	EC-EARTH	CLMcom-CCLM4-8-17	r12i1p1	met_21
			GERICS-REMO2015	r12i1p1	met_23
			KNMI-RACMO22E	r12i1p1	met_24
				r1i1p1	met_27
				r3i1p1	met_30
			SMHI-RCA4	r12i1p1	met_25
				r1i1p1	met_28
				r3i1p1	met_31
	IPSL	IPSL-CM5A-MR	IPSL-WRF381P	r1i1p1	met_35
			KNMI-RACMO22E	r1i1p1	met_36
			SMHI-RCA4	r1i1p1	met_37
	MIROC	MIROC5	CLMcom-CCLM4-8-17	r1i1p1	met_40
			GERICS-REMO2015	r1i1p1	met_41
	MOHC	HadGEM2-ES	CLMcom-CCLM4-8-17	r1i1p1	met_50
			CNRM-ALADIN63	r1i1p1	met_51
			GERICS-REMO2015	r1i1p1	met_53
			ICTP-RegCM4-6	r1i1p1	met_54
			IPSL-WRF381P	r1i1p1	met_55
			KNMI-RACMO22E	r1i1p1	met_56
			MOHC-HadREM3-GA7-05	r1i1p1	met_57
			SMHI-RCA4	r1i1p1	met_58
	MPI-M	MPI-ESM-LR	CLMcom-CCLM4-8-17	r1i1p1	met_67
			CLMcom-ETH-COSMO-crCLIM-v1-1	r1i1p1	met_68
				r2i1p1	met_74
			GERICS-REMO2015	r3i1p1	met_77
			ICTP-RegCM4-6	r1i1p1	met_70
			KNMI-RACMO22E	r1i1p1	met_71
			MPI-CSC-REMO2009	r1i1p1	met_72
				r2i1p1	met_75
			SMHI-RCA4	r1i1p1	met_73
				r2i1p1	met_76
				r3i1p1	met_78
	NCC	NorESM1-M	CLMcom-ETH-COSMO-crCLIM-v1-1	r1i1p1	met_82
			GERICS-REMO2015	r1i1p1	met_84
			IPSL-WRF381P	r1i1p1	met_85
			KNMI-RACMO22E	r1i1p1	met_86
			SMHI-RCA4	r1i1p1	met_87

All realisations can be assigned to an RCP^30^. Institutional (inst) regional climate models (inst.rcm) were run, coupled with a global climate model (gcm). The realisation indicates the initial starting situation and the met_id is the final identification.

The simulation data have a spatial resolution of 0.11° (12.5 km) and are bias-adjusted using the trend-preserving ISIMIP3BASD methodology^32^. An ensemble version (v20.0e)^29^ of gridded E-OBS data^33^ with a different gridding method and improved uncertainty estimate served as reference data for the bias-adjustment. During bias-adjustment the realisation data (HI-CAM) are aligned with the statistics of the reference data. The time period considered here is 1971 to 2000. They include daily minimum, maximum and average temperature as well as daily precipitation. The E-OBS data^33^ were derived based on climate stations across Europe and tested thoroughly for their quality^29,34^. The underlying climate station data vary in their temporal and spatial coverage across Europe and carry some inhomogeneities. Consequently, the gridded E-OBS data also exhibit inhomogeneities^34^. E-OBS data users of any version have to be aware of these limitations. The ensemble version v20.0e was remapped from their original resolution 0.25° (25 km) to 0.11° using a conservative remapping method to fit the realisation data (HI-CAM) resolution.

The bias-adjusted realisation data are then spatially disaggregated from 0.11° to 0.03125° (3 km) using External Drift Kriging (v2.0.0)^35^ with elevation^36^ as external drift factor. Realisation variables with daily temporal resolution considered for the bioclimatic calculation are as follows: daily average air temperature (tas) [°C]^37^, maximum air temperature (tasmax) [°C]^38^, minimum air temperature (tasmin) [°C]^39^ and precipitation (pr) [mm d^−1^]^40^. UFZ mHM-Hydrological model output^41–43^ daily potential evapotranspiration (pet) [mm d^−1^]^44^ and weekly soil water content for 6 different soil layers (swc) [mm] serve as additional input. The realisation data and mHM model output are available in the WGS84 reference frame (EPSG: 4326) with a spatial resolution of 0.03125° for the extent of the European continent excluding Turkey, Iceland and Russia (lat: 35° lon: −11°, lat: 71.5° lon: 41°) and temporal range from 1971 to 2098, Fig. 1. We chose to use biogeographical regions of Europe (EBR)^45^ to facilitate the presentation of bioclimatic variables in the following.

**Fig. 1:**
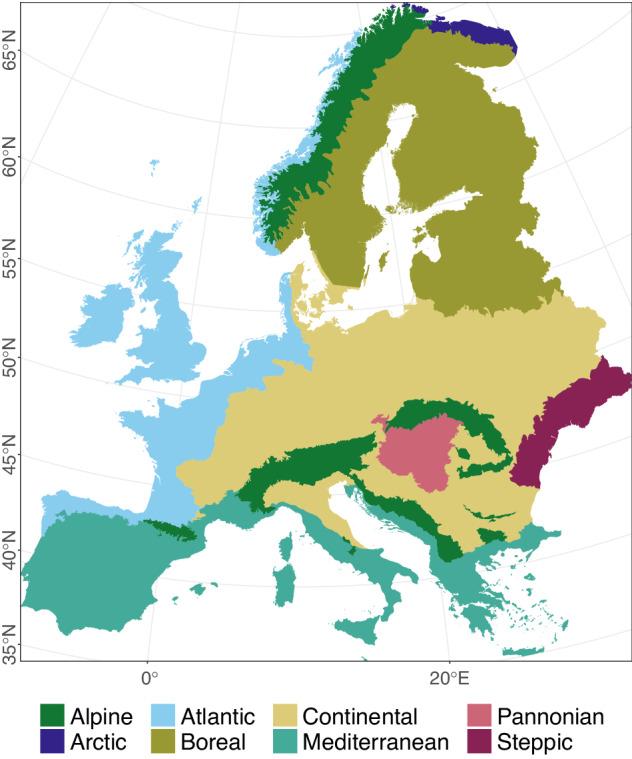
HI-CAM data availability for Europe. Dividing the area in eight biogeographical regions of Europe (EBR)^45^ facilitates the presentation of bioclimatic variables in selected subsequent figures.

### Calculation of bioclimatic variables

**Fig. 2:**
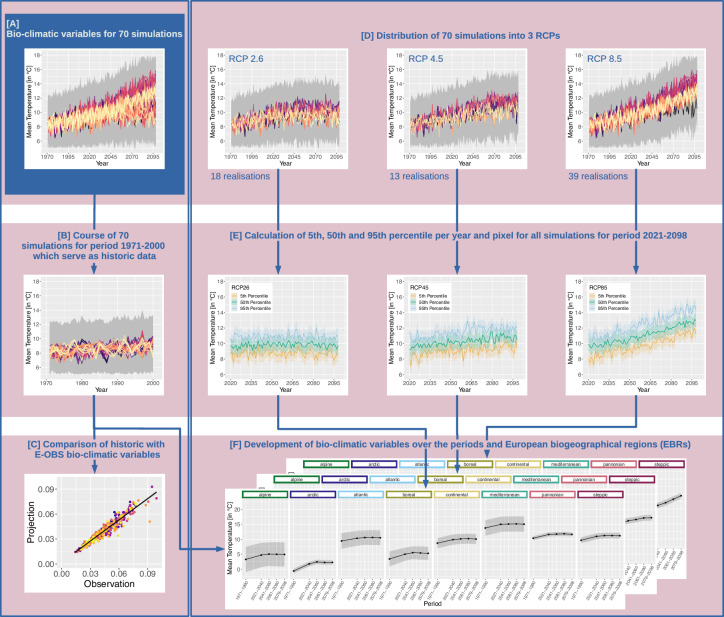
The process of bioclimatic variable calculation for historical 1971 to 2000 and projection period 2021 to 2098 outlined by a flowchart. Capital letters indicate the steps and are referred to in the following descriptions. The total of 70 HI-CAM^28^ EURO-CORDEX^24–26^ CMIP5^19^ climate realisations serve as input [**A**]. We calculated the historical period based on the median of all realisations [**B**] and validated them against E-OBS version (v20.0e)^29^ [**C**]. In addition, the realisations can be assigned to three different RCPs (Representative Concentration Pathways) − 2.6, 4.5, 8.5 [**D**]. Here, we extracted the 5^th^, 50^th^ and 95^th^ percentile within each RCP [**E**] to account for the variance within. From all periods (historic and projection) we have then extracted the temporal median [**F**].

Figure 2 illustrates the process of bioclimatic variable computation using capital letters within the following subsections. A total of 26 bioclimatic variables were selected following these of ANUCLIM^46^ and *WorldClim* variables^7,8^ as well as literature review, see Table 2. The variables tas, tasmax, tasmin, pr, pet and swc serve as input to the calculation. For swc the sum over all soil layers per time step per realisation was calculated beforehand. We utilised anuclim and atmos indicators from Python package *xclim*^47^ as well as algorithms of our own for the calculation. We calculated the bioclimatic variables for each realisation with a temporal sampling period of 1 year for the time range 1971 to 2000 serving as historic and 2021 to 2098 serving as projection time period. This accounts for 26 bioclimatic variables for 70 realisations [A]. The following descriptions apply pixel-wise to each bioclimatic variable.

**Table 2:** 26 calculated bioclimatic variables with short and full bioclimatic variable names as well as units the variables are provided in.

Variable name	bioclimatic variable	Unit	Name in Worldclim
BioVar1	Mean Temperature	C°	BIO1
BioVar2	Diurnal Temperature Range (dtr)	C°	BIO2
BioVar3	Isothermality (dtr/(tmax-tmin))	%	BIO3
BioVar4	Temperature Seasonality (SD * 100)	%	BIO4
BioVar5	Max Temperature of the Warmest Month	C°	BIO5
BioVar6	Min Temperature of the Coldest Month	C°	BIO6
BioVar7	Temperature range	C°	BIO7
BioVar8	Mean Temperature of the Wettest Quarter	C°	BIO8
BioVar9	Mean Temperature of the Driest Quarter	C°	BIO9
BioVar10	Mean Temperature of the Warmest Quarter	C°	BIO10
BioVar11	Mean Temperature of the Coldest Quarter	C°	BIO11
BioVar12	Precipitation Sum	mm	BIO12
BioVar13	Index of Aridity (tavg July/annual pre)	unitless	
BioVar14	Precipitation Range	mm	
BioVar15	Precipitation Seasonality (mean/SD*100)	%	BIO15
BioVar16	Precipitation of the Wettest Quarter	mm	BIO16
BioVar17	Precipitation of the Driest Quarter	mm	BIO17
BioVar18	Precipitation of the Warmest Quarter	mm	BIO18
BioVar19	Precipitation of the Coldest Quarter	mm	BIO19
BioVar20	Last Spring Frost	DOY	
BioVar21	Growing Degree Days above 5°C	K days	
BioVar22	Continentality Index^55^	unitless	
BioVar23	Dry Days	No. of days	
BioVar24	Max Consecutive Dry Days	No. of days	
BioVar25	Water Deficit-inverted^56^	mm	
BioVar26	Mean Soil Water Content all Soil Layers	mm	

For comparison names of similarly/identical computed variables of well known *WorldClim*^7,8^ and ANUCLIM^46^ are listed in the far right columnn.

### Calculation of ensemble statistics

The time range 1971 to 2000 serves as historic time period due to data availability not before 1971. Over the course of the period all realisations run quite similar [B]. We extracted the temporal 5^th^, 50^th^ and 95^th^ percentile per realisation to receive three values representing the time period [F]. This serves as input to our data validation [C].

For the projection time period, we selected 4 time periods with a similar range to the *WorldClim*^8^ data, which are common predictor variables in species distribution modelling studies. The periods are as follows 2021 to 2040, 2041 to 2060, 2061 to 2080 and 2079 to 2098 due to data availability until 2098 and to keep the length of 20 years consistent. Here we extracted two versions for the bioclimatic variables.

**Fig. 3:**
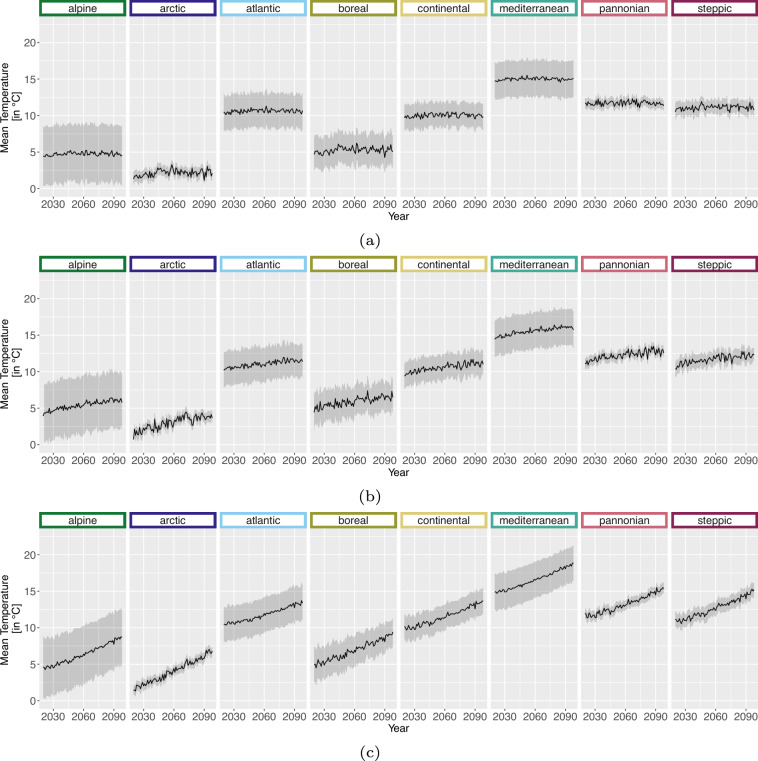
Annual course of BioVar1 (Mean Temperature) 50^th^ percentile for eight EBRs^45^ during the period 2020 to 2098. Here the mean (black line) and standard deviation (grey background) is extracted per EBR^45^. Subfigures show annual mean temperature for RCP 2.6 (**a**), 4.5 (**b**) and 8.5 (**c**).

**Fig. 4:**
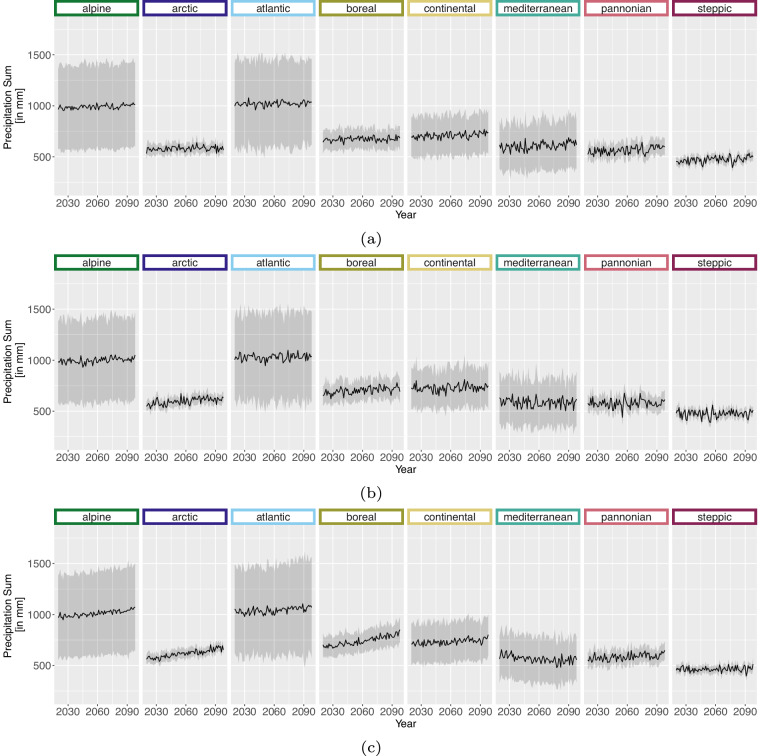
Annual course of BioVar12 (Precipitation Sum) 50^th^ percentile for eight EBRs^45^ during the period 2020 to 2098. Here the mean (black line) and standard deviation (grey background) is extracted per EBR^45^. Subfigures show BioVar12 for RCP 2.6 (**a**), 4.5 (**b**) and 8.5 (**c**).

**Fig. 5:**
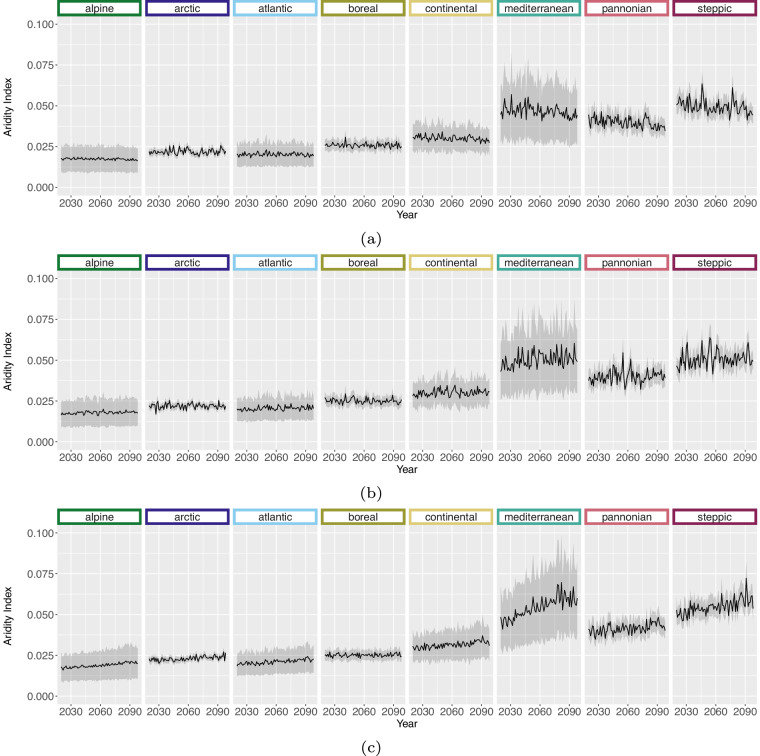
Annual course of BioVar13 (Index of Aridity) 50^th^ percentile for eight EBRs^45^ during the period 2020 to 2098. Here the mean (black line) and standard deviation (grey background) is extracted per EBR^45^. Subfigures show BioVar13 for RCP 2.6 (**a**), 4.5 (**b**) and 8.5 (**c**).

**Fig. 6:**
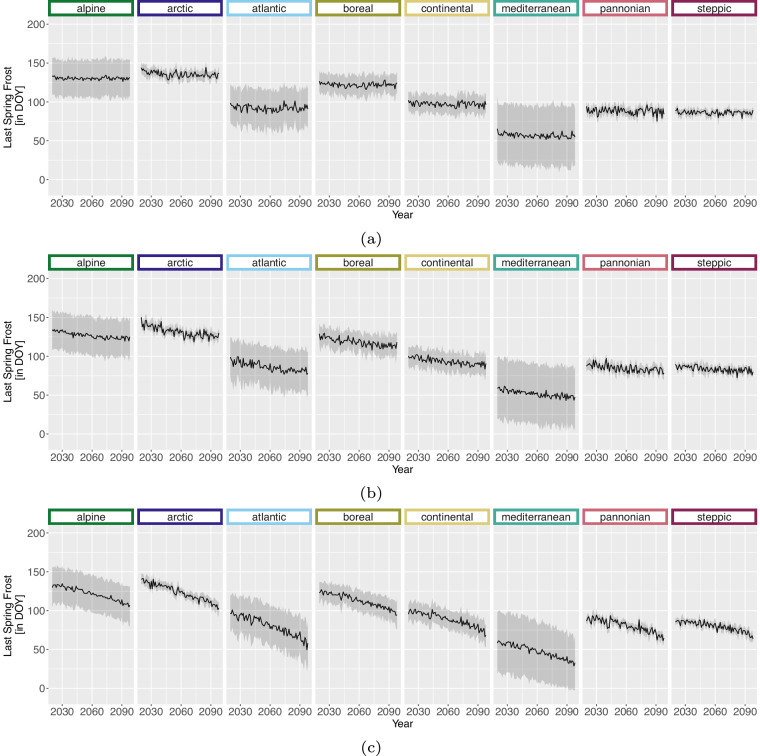
Annual course of BioVar20 (Last Spring Frost) 50^th^ percentile for eight EBRs^45^ during the period 2020 to 2098. Here the mean (black line) and standard deviation (grey background) is extracted per EBR^45^. Subfigures show BioVar20 for RCP 2.6 (**a**), 4.5 (**b**) and 8.5 (**c**).

**Fig. 7:**
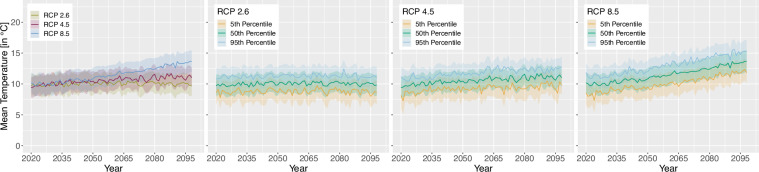
BioVar1 (Mean Temperature) for all RCPs (left), RCP 2.6, RCP 4.5 and RCP 8.5. Figures of single RCPs include the 5^th^, 50^th^ an 95^th^ percentiles. For all subfigures the mean (thick line) and standard deviation (shaded background) for continental EBR are extracted.

**Fig. 8:**
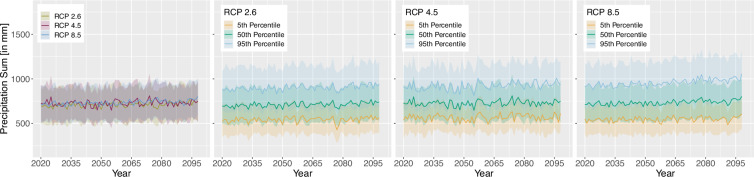
BioVar12 (Precipitation Sum) for all RCPs (left), RCP 2.6, RCP 4.5 and RCP 8.5. Figures of single RCPs include the 5^th^, 50^th^ an 95^th^ percentiles. For all subfigures the mean (thick line) and standard deviation (shaded background) for continental EBR are extracted.

**Fig. 9:**
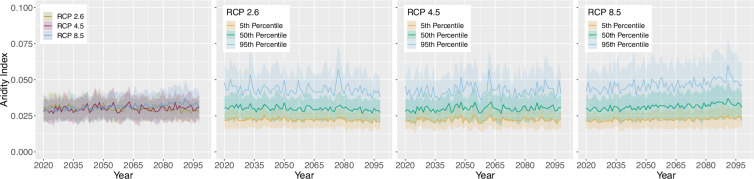
BioVar13 (Index of Aridity) for all RCPs (left), RCP 2.6, RCP 4.5 and RCP 8.5. Figures of single RCPs include the 5^th^, 50^th^ an 95^th^ percentiles. For all subfigures the mean (thick line) and standard deviation (shaded background) for continental EBR are extracted.

1. As we can assign each realisation to an RCP [D], Table 1, we extracted the 50^th^ but also 5^th^ and 95^th^ percentile from all realisations belonging to the same RCP [E] to obtain the variance within. The annual 50^th^ percentile development of selected variables per EBR is shown in Figs. 3, 4, 5 and 6. In addition, Figs. 7, 8, 9 and 10 reveal the annual course of the 5^th^, 50^th^ and 95^th^ percentile for the continental EBR as an example. Subsequently, we extracted the temporal 5^th^, 50^th^ and 95^th^ percentile for each RCP and period similar to the historic data [F]. This results in 4 periods and 3 RCPs each accompanied by 9 percentiles (within RCP and temporal 5^th^, 50^th^, 95^th^).

**Fig. 10:**
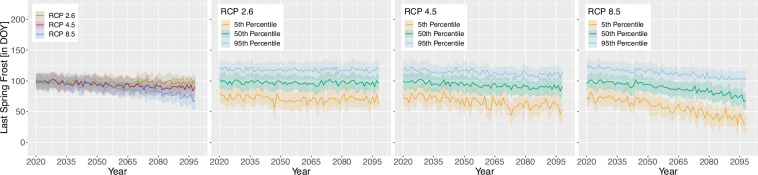
BioVar20 (Last Spring Frost) for all RCPs (left), RCP 2.6, RCP 4.5 and RCP 8.5. Figures of single RCPs include the 5^th^, 50^th^ an 95^th^ percentiles. For all subfigures the mean (thick line) and standard deviation (shaded background) for continental EBR are extracted.
2. In addition, we have extracted the temporal 5^th^, 50^th^ and 95^th^ percentile for each period (realisation and projection). This results in 4 periods and 3 percentiles, for all 70 realisations and allows the user to chose only realisations of interest for further analysis. An example of the temporal course of BioVar1 (Mean Temperature) for the realisation (met) 46 is included in Fig. 11.

**Fig. 11:**
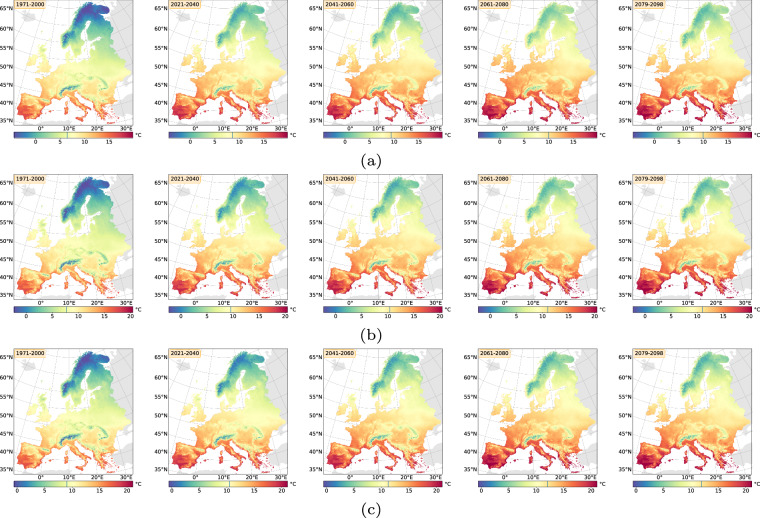
Temporal course over all 5 time steps (historic and projections) of BioVar1 - Mean Temperature - for realisation (met) 46, which is defined within RCP 4.5. Subfigure (**a**) shows the 5^th^, (**b**) the 50^th^ and (**c**) the 95^th^ temporal percentile per time step. The blue line within the legend indicates the areal mean.

All described calculations were performed using Python package *xarray*^48^. We re-projected the final data products to ETRS89 Lambert Azimuthal Equal Area (EPSG: 3035) with resampling method nearest neighbour using *rioxarray*^49^ and set the spatial resolution to 3 km.

## Data Records

The dataset can be obtained from a Zenodo repository^50^ which is comprised of ten tar.gz files.

Each file inside the tar.gz contains all 26 bioclimatic variables with order and names according to Table 2 column *Variable name*. The filenames for the realisations are described as follows:

bioVars_1971–2000_met.tar.gz → Projections per realisations for period 1971–2000

bioVars_2021–2040_met.tar.gz → Projections per realisations for period 2021–2040

bioVars_2041–2060_met.tar.gz → Projections per realisations for period 2041–2060

bioVars_2061–2080_met.tar.gz → Projections per realisations for period 2061–2080

bioVars_2079–2098_met.tar.gz → Projections per realisations for period 2079–2098

Regarding the reference and projection periods the filenames inside the tar.gz files are

**biovars_met** ***01*** **_** ***1971–2000*** **_** ***5*** **.tif**

with ***01*** as realisation number, ***1971–2000*** as period and ***5*** as temporal percentile within the period.

For bioVars separated into RCPs the filenames are described as follows:

bioVars_2021–2040_rcp.tar.gz → Projections per RCP for period 2021–2040

bioVars_2041–2060_rcp.tar.gz → Projections per RCP for period 2041–2060

bioVars_2061–2080_rcp.tar.gz → Projections per RCP for period 2061–2080

bioVars_2079–2098_rcp.tar.gz → Projections per RCP for period 2079–2098

Regarding the RCP for the projection periods the filenames inside the tar.gz files are

**biovars_rcp** ***2.6*** **_** ***50*** **perc_** ***2061–2080*** **_** ***5*** **.tif**

with ***2.6*** as RCP, ***50*** as percentile within the RCP, ***2061–2080*** as period and ***5*** as temporal percentile within the period.

In addition, the validation results are part of the Zenodo repository^50^. The folders *eobs* and *worldclim* each contain scatter plots of all possible validation variables (folder *plots*) as well as statistics (folder *stats*) for R^2^ and RMSE.

## Technical Validation

**Fig. 12:**
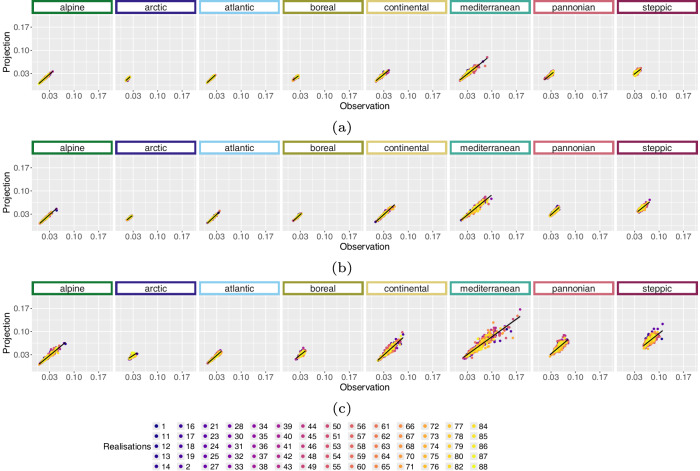
Scatterplots of E-OBS version (v20.0e)^29^ vs. historical bioclimatic BioVar13 (Index of Aridity) for the time period 1971 to 2000 and each realisation separately. The comparison was iterated 20 times for 10 randomly sampled pixel per EBR^45^. These plots include 5 randomly sampled pixel per realisation from within the comparison data pool. Subplots indicate the temporal (**a**) 5^th^, (**b**) 50^th^ and (**c**) 95^th^ percentile.

**Fig. 13:**
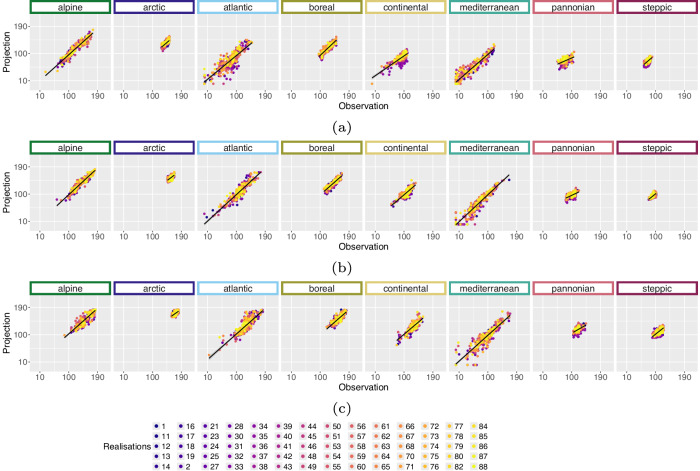
Scatterplots of E-OBS version (v20.0e)^29^ vs. historical bioclimatic BioVar20 (Last Spring Frost) for the time period 1971 to 2000 and each realisation separately. The comparison was iterated 20 times for 10 randomly sampled pixel per EBR^45^. These plots include 5 randomly sampled pixel per realisation from within the comparison data pool. Subplots indicate the temporal (**a**) 5^th^, (**b**) 50^th^ and (**c**) 95^th^ percentile.

We applied the bioclimatic calculation including the temporal 5^th^, 50^th^ and 95^th^ percentile on the E-OBS data to compare them with our historical bioclimatic variables based on HI-CAM data. Similarly, these E-OBS bioclimatic variables were reprojected to ETRS89 Lambert Azimuthal Equal Area (EPSG: 3035) using nearest neighbour as resampling method and the spatial resolution was set to 3 km. As the variables potential evaporation and soil water content are not available from E-OBS, we omitted bioclimatic variables that rely on these variables, which are Mean Soil Water Content and Water Deficit. We compared our historic variables with E-OBS variables based on the period percentiles resolution (selection of scatter plots, Figs. 12, 13). A quantitative analysis was run on the period percentiles resolution. In this quantitative analysis, we randomly sampled 10 pixel values per entity which is variable, temporal percentile and EBR from E-OBS as well as variable, realisation, temporal percentile and EBR from HI-CAM bioclimatic variables. For each of these entities we calculated the R^2^ and RMSE, see tables in Zenodo repository^50^. This process was iterated 20 times and statistics were averaged among them. We see highest R^2^ values for bioclimatic variables without precipitation involved (e.g. Mean Temperature - BioVar1, Diurnal Temperature Range - BioVar2, Mean Temperature of the Warmest Quarter - BioVar10, Mean Temperature of the Coldest Quarter - BioVar11 and Growing Degree Days above 5 °C - BioVar21 - please see Zenodo repository^50^ for these plots). Two rather extreme event bioclimatic variables Dry Days - BioVar23 and Max Consecutive Dry Days - BioVar24 - please see Zenodo repository^50^ for these plots) show lowest agreement across all EBRs. The three smallest EBRs (Arctic, Pannonian and Steppic) show lower agreement with E-OBS variables than other EBRs. The origin of this lower agreement might evolve from lower station density in these areas (Spain, the Balkans and Northern Scandinavia) that were used for generating the E-OBS data^34^. The lower density in Spain, which is largely part of EBR Mediterranean, affects the agreement less as it does in the low station density EBRs that are much smaller (Pannonian and Steppic in the Balkans as well as Arctic in Northern Scandinavia).

As the bias-correction of the HICAM variables was done using the E-OBS v20.0^29^ data, we are confident, that a change to the newest E-OBS v30.0^29^ data for the validation might not improve the data to a significant amount. E-OBS v20.0 data were used in recent publications and proofs to be a well accepted standard (among other^51,52^). The newest E-OBS version (v30.0)^29^ offers a longer time-frame, more variables at higher spatial resolution as well as an enlarged station density in comparison to E-OBS v20.0^29,53^. Merely, the enlarged station density might improve our bioclimatic variables from the process of bias-correction. However, our study was designed and conducted using E-OBS v20.0^29^ for bias-correction, ensuring consistency in data analysis, validation and results interpretation. Changing to E-OBS v30.0^29^ might introduce discrepancies in results due to changes in data handling, resolution or underlying corrections.

To rule out lower qualitative biolimatic variables due to lower station density in E-OBS v20.0^29^ and overoptimistic validation using the same data for bias-correction as well as validation, we enlarged the validation using  *WorldClim* data version 2.1^8^. We utilised the data with 2.5 minutes spatial resolution, re-projected and resampled these data to EPSG:3035 and 3 km to match our bioclimatic variables. As *WorldClim*^8^ offers the temporal mean per time period, we used our 50^th^ temporal percentile data for validation. We were able to validate 17 of our 26 bioclimatic variables, that are available from *WorldClim*^8^ as well.

Similar to the validation using E-OBS^29^ data, we randomly sampled 20 pixel values per variable and EBR from *WorldClim*^8^ as well as variable, realisation and EBR from HI-CAM bioclimatic variables. The amount was increased to account for slightly shifted pixel locations between both inputs. Again, this process was iterated 20 times and statistics were averaged among them.

**Fig. 14:**
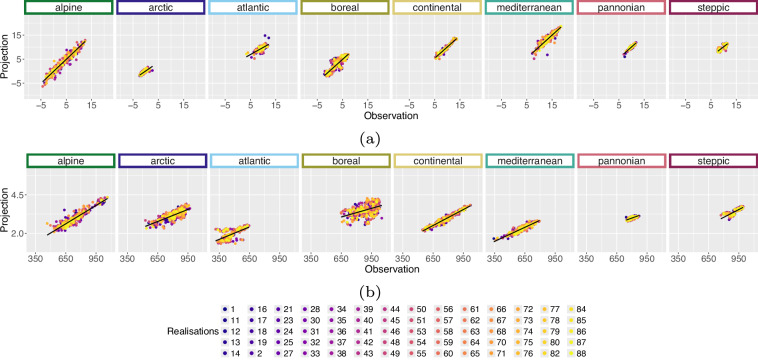
Scatterplots of *WorldClim* version (2.1)^8^ vs. historical bioclimatic variables for the time period 1971 to 2000 for each realisation separately. The comparison was iterated 20 times for 20 randomly sampled pixel per EBR^45^. These plots include 5 randomly sampled pixel per realisation from within the comparison data pool. Subplots indicate the (**a**) BioVar1 (Mean Temperature) and (**b**) BioVar4 (Temperature Seasonality). The axes limits vary in result of different calculation of *WorldClim* and historical Temperature Seasonality.

**Fig. 15:**
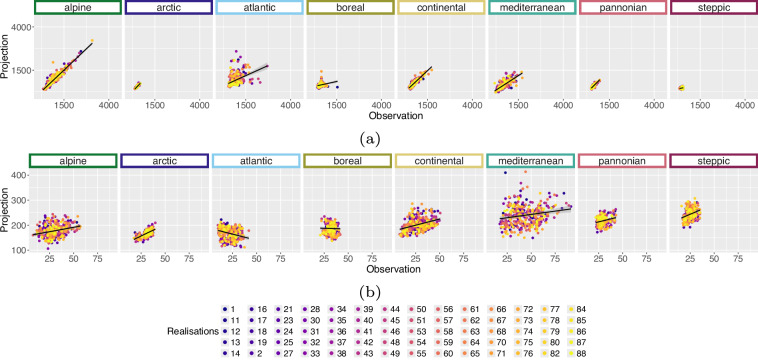
Scatterplots of *WorldClim* version (2.1)^8^ vs. historical bioclimatic variables for the time period 1971 to 2000 for each realisation separately. The comparison was iterated 20 times for 20 randomly sampled pixel per EBR^45^. These plots include 5 randomly sampled pixel per realisation from within the comparison data pool. Subplots indicate the (**a**) BioVar12 (Precipitation Sum) and (**b**) BioVar15 (Precipitation Seasonality). The axes limits vary in result of different calculation of *WorldClim* and historical Precipitation Seasonality.

The results for BioVar1 (Mean Temperature) and BioVar4 (Temperature Seasonality), see Fig. 14, as well as BioVar12 (Precipitation Sum) and BioVar15 (Precipitation Seasonality), Fig. 15, remain very promising, see statistics in the Zenodo repository^50^. Some variables from *WorldClim* version 2.1^8^ show a slightly different methodology of extraction (Temperature Seasonality – BioVar4, Diurnal Temperature Range - BioVar2), which results in higher RMSE values, see plots and statistics in the Zenodo repository^50^. Partly unfavourable R^2^ values (BioVar2) e.g. for Boreal EBR might evolve from the fact, that *WorldClim*^8^ variables are averaged for the time period 1970–2000 whereas for BioVars the 50^th^ percentile is utilised for the time period 1971–2000. Especially for extreme variables with extreme seasonal climatic differences (e.g. EBR Boreal) this can lead to unfavourable R^2^ results.

## Usage Notes

The bioclimatic data are provided here in three parts. (1) The historic time period, which is derived for the time period 1971 to 2000 per realisation. Whereas for the projection period (2) the realisations are then separated into three RCPs and combined within. Here the combining encompasses the 5^th^, 50^th^, 95^th^ percentile which allows to account for the variance among the realisations within the RCP. If only specific realisations are to be taken into account (3) we provide all realisations separately. All three parts are supplied as the temporal 5^th^, 50^th^, 95^th^ percentile for predefined periods as described in the Method section. We are encouraged to define similar temporal periods (historic as well as projection time periods) as in *WorldClim*^8^. Providing the historical and the projection periods derived from the same simulation data enables the usage of these bioclimatic variables with low shifting bias among. Use cases for these variables might be species distribution modelling. The variables from the historic period act as predictor variables during the model training process and the variables from the projection periods (RCP or singular realisations) serve as predictor variables for the model projection. A principal component analysis (PCA) or a correlation analysis of all variables within the historic period reveals a high correlation of multiple variables and should be considered for further use of the data. Despite the utilisation of E-OBS v20.0^29^ (released October 2019) instead of E-OBS v30.0^29^ (released September 2024) for bias-correction and validation, we are confident about the quality of our bioclimatic variables. Nevertheless, areas of more sparse station coverage between both E-OBS versions might introduce some slight uncertainties in our bioclimatic variables in those regions.

## Data Availability

The code, we generated the bioclimatic variables with, can be accessed under the zenodo repository^54^ Python packages and versions are listed within the requirements.txt file.
